# Allowing access to parents/caregivers into COVID-19 hospitalization areas does not increase infections among health personnel in a pediatric hospital

**DOI:** 10.3389/fped.2022.896083

**Published:** 2022-09-14

**Authors:** Daniela De la Rosa-Zamboni, María José Adame-Vivanco, Mercedes Luque-Coqui, Carlos Mauricio Jaramillo-Esparza, Fernando Ortega-Riosvelasco, Irineo Reyna-Trinidad, Ana Carmen Guerrero-Díaz, Sergio Gabriel Ortega-Ruiz, Sergio Saldívar-Salazar, Mónica Villa-Guillen, Jaime Nieto-Zermeño, Sergio René Bonilla-Pellegrini, Lourdes María del Carmen Jamaica Balderas

**Affiliations:** ^1^Department of Comprehensive Patient Care, Hospital Infantil de México Federico Gómez, Mexico City, Mexico; ^2^Pediatric Psychology Coordination, Hospital Infantil de México Federico Gómez, Mexico City, Mexico; ^3^Hospital Infantil de México Federico Gómez, COVID-19 Area, Mexico City, Mexico; ^4^Department of Epidemiology, Hospital Infantil de México Federico Gómez, Mexico City, Mexico; ^5^Department of Nursing, Hospital Infantil de México Federico Gómez, Mexico City, Mexico; ^6^Department of Psychology, Hospital Infantil de México Federico Gómez, Mexico City, Mexico; ^7^Hospital Infantil de México Federico Gómez, Mexico City, Mexico

**Keywords:** hospital transmission, SARS – CoV – 2, pediatric, children, visitation, Latin America, hospital workers, hospital acquired SARS-CoV-2

## Abstract

**Background:**

At the beginning of the current COVID-19 pandemic, it became critical to isolate all infected patients, regardless of their age. Isolating children has a negative effect on both, them and their parents/caregivers. Nevertheless isolation was mandatory because of the potential risk that visitation might have on COVID-19 dissemination mostly among health personnel.

**Methods:**

From the starting of the COVID-19 pandemic in our pediatric hospital visits were forbidden. This 2 months period (April–May) was called P1. In June parents were allowed to visit (P2), under a visiting protocol previously published. Hospital workers were monitored for the presence of COVID-19 symptoms and tested for the infection when clinically justified. The positivity proportion and the relative risk (RR) of COVID-19 among the health personnel between periods were calculated. The caregivers were also followed up by phone calls.

**Results:**

Since April 2020 to November 2020, 2,884 health personnel were studied for 234 days, (318,146 workers days). Although the COVID-19/1,000 health personnel days rate decreased from one period to another (1.43 vs 1.23), no statistically significant differences were found. During P1, 16 patients with COVID-19 were treated. During the follow up none of the family members were infected/symptomatic in P1, while in P2, 6/129 (4.65%) were symptomatic or had a positive test. All of them initiated between 2 and 4 days after the patient's admission. As they also had some other infected family members it was not possible to ensure the source of infection. There were no statistically significant differences in the RR of COVID-19 in health personnel, (RR 1, 95% CI 0.69–1.06, *p* = 0.162).

**Conclusions:**

When safely implemented, allowing parents/caregivers to spend time with their hospitalized COVID-19 children does not increase the contagion risk for hospital workers or among themselves.

## Introduction

In February 2020, the first COVID-19 case was registered in Mexico (1). Mexico City has a total population of 8,855,000 inhabitants. As of this day, there have been 166,517 (83,324 female and 83,193 male) total cases of COVID-19 in the population group of those younger than 20 years old of a total of 1,427,083 cumulative confirmed cases, which represents the 1.8% of the total population. Similar than the literature reported in other countries. COVID-19 incidence in children younger than 20 years, especially in those younger than 10 years, is several times lower than in adults. The interpretation of these results is limited by the quality of the information provided by the General Epidemiology Direction of the Mexican Ministry of Health, leading to a sub-estimation of cases because of the poor testing in the country (2, 3).

In late March 2020, hospital isolation protocols were started for infection containment and to protect the hospital health personnel, which are at an increased risk of contracting COVID-19 (4–7). Epidemiological surveillance was started to track possible infections from the detected cases.

The entry of visitors to the COVID areas was restricted; children's parents/caregivers with COVID-19 were not allowed in to see their children. However, the harmful effects on mental health in hospitalized patients have been well documented (8), and this extends to the COVID-19 adult population as well (9, 10). Even when the repercussions of isolation have been mostly assessed in adult population, several authors have documented the effects that hospital isolation has on children with COVID-19 and their families (11–15). Some of the effects seen in parents/caregivers with children infected with COVID-19 are anxiety, depression, and dream anxiety (13), as well as sadness, anger and suffering which can lead to further post-traumatic stress disorder (14).

These negative effects, as a consequence of the separation, motivated our hospital to allow the parents/caregivers to enter to COVID-19 areas. Although it is known that in pediatric hospitals, children are not the main drivers of transmission (16, 17), and most cases among hospital workers are transmitted from coworkers (6, 7, 16–18); we were still concerned about the safety of allowing visits.

In this study we analyze the COVID-19 contagiousness risk in health personnel and patients' close family members with the implementation of a protocol that allowed extended visits to hospitalized children in a setting in which the visits were initially restricted.

## Materials and methods

The “Hospital Infantil de México Federico Gómez” (Mexico Children's Hospital “Federico Gómez” – HIMFG –) is a national reference, tertiary, teaching institute for pediatric care in Mexico City. It has 290 hospital beds, two intensive care units, and a neonatal intensive care unit. In the hospital, “COVID areas” were adapted for patients diagnosed with COVID-19 by RT-PCR. The conversion of the hospital to a COVID hospital was described by Villa-Guillen et al. Broadly speaking, the different areas that were adapted within the hospital had 10 intensive care beds, five neonatal intensive care beds, 28 beds for non-critical patients and 14 beds within the emergency room for patients with suspected COVID-19. It is important to mention that these areas do not have individual rooms and the beds are not divided from one another. The ventilation of these areas is natural and air extractors were placed (19). Visitors' access to these areas was restricted from April to June 2020; which was period 1 of the study. Period 2 began in June 2020, when visits were permitted, following the protocol described by Luque-Coqui et al. (20). An instruction sheet was given to the “family members of hospitalized patients, highlighting the role of the caregiver (the tasks that must be performed for nursing support and the COVID area rules), and the adherence to hygiene and safety rules” (20). One visitor per patient allowed regardless of the COVID area the patient was in. Visitors were allowed for 6 h per day if patients were at least 6 years old. Family members of younger patients were allowed for 8 h per day.

Children's caregivers were carefully selected: first, it was verified that they did not have COVID-19 symptoms; if they did, they were sent home and another caregiver was sought. Not all parents/caregivers underwent RT-PCR testing before coming in to visit. As described in the protocol by Luque et al., initially it was intended to perform RT-PCR tests on all parents/caregivers, but given the high frequency of false-negative results in the first days of infection, and false-positives 8 days after infection, it was decided only to test parents/caregivers that met the definition of contact. Also there were not enough resources to do test every parent/caregiver. Caregivers with no symptoms and with 6–8 days from having contact with a COVID-19 patient underwent a RT-PCR test. If the result was positive another caregiver was sought; if it was negative, they underwent a psychological test to assess whether they could tolerate isolation. The test that was applied was the Goldberg General Health Questionnaire, which has already been validated for use in the Mexican population (21). Parents/caregivers were given training on the risks of contracting COVID-19, on the use of PPE, on how to give necessary care to the patient and on other protective measures such as hand hygiene. Regarding PPE, visitors had to use KN95 masks, face shields, gloves, disposable gowns and disposable boots. Personal and telephone follow-up was carried out with the family member in charge in order to provide psychological and emotional support tools. This same group of researchers has previously described the implementation of security measures to allow visitors to COVID areas (20).

Simultaneously, the source of COVID-19 infections among health personnel was being traced within the hospital. Contacts were defined according to CDC criteria (22–24), as those who had been with an infected person 2 days before the onset of symptoms or 2 days before the positive test was recorded and until the time the infected person had been isolated. Contact tracing was performed on all contacts and all underwent RT-PCR. Detected cases and contacts were asked to stay at home for 2 weeks, during which they were contacted by phone by the Epidemiology Department to evaluate their clinical evolution. All patients with temperature >38°C, anosmia, or SpO_2_ >90% were considered as positive, regardless of the RT-PCR result. The epidemiological surveillance of all cases and contacts was carried out from April 2020 to November 2020. The tracing methodology within our hospital was published by de la Rosa-Zamboni et al. (25).

In addition, we performed a sub-analysis of the family members. They were interrogated about symptoms and signs suggestive of COVID-19 infection, or PCR testing within 14 days of hospital stay. The signs and symptoms considered suggestive were: dysgeusia, arthralgia, myalgia, dyspnea, pharyngodynia, shivers, rhinorrhea, conjunctivitis, chest pain, severe fatigue, cough, fever >38°C, and anosmia. They were also questioned about whether any tests were performed to confirm the diagnosis.

### Statistical analysis

We conducted the statistical analysis using SPSS 21. Kolmogorov–Smirnov tests were used to assess data distribution. Given that the quantitative variables did not have a normal distribution, median and interquartile ranges were used for the descriptive analysis; frequencies and percentages described the qualitative variables. To compare the positivity proportion of COVID-19 before and after the caregivers were allowed to visit, chi squared tests were used. Relative risk (RR) was estimated to evaluate the risk of being infected when allowing parents/caregivers to visit and getting infected with COVID-19. A *p*-value < 0.05 was considered statistically significant. The rate of health personnel who underwent RT-PCR per 1,000 patient days was calculated, as well as the rate of positive cases for COVID-19 of health personnel per 1,000 patient days.

## Results

Since April 2020 to November 2020, 2,884 health personnel were studied for 234 days, which correspond to 318,146 workers days. The study was divided in two time periods: The first one in which the parents/caregivers still could not enter to visit their children, which consisted of 57 days (77,497.2 worker-days). The second period started once parents/caregivers were allowed to visit, and it comprised 177 days (240,649.2 worker-days). From the 2,884 health personnel, 158 belonged to COVID areas. The first period had 4,245.7 shifts per worker and the second one 13,484.

During the time of the study 1,659 health personnel underwent RT-PCR, of which 406 were positive (24.47%). In the first period, 301 RT-PCR tests were carried out, of which 111 were positive (36.88%); in the second period, 1,358 people were tested and 295 were positive (21.72%). During the first period 36 health personnel were tested and 10 were positive (27.78%), while in the second period 188 health personnel took the test and 24 were positive (15.18%). Table 1 shows the positivity rates as well as the relative risk during the first and second periods.

**Table 1:** RT-PCR tests, positivity rates and relative risk during the first and second period.

		**Total**	**1 st period patients without caregivers**	**2 nd period patients with caregivers**	*p-*Value*
All health personnel (*N* = 2,884)	HP tested No.	1,659	301	1,358	–
	Positive cases No. (%)	406 (24.47)	111 (36.88)	295 (21.72)	<0.0001
	Rate of HP tested/1,000 HP days	2.18	2.4	2.13	
	RR (CI_95%_)	–	1	0.86 (0.69–1.06)	0.162
	COVID-19 rate per 1,000 HP days	1.28	1.43	1.23	
	RR (CI_95%_)	–	1	1 (0.69–1.06)	0.162
Health personnel from COVID-areas, (*N* = 158)	HP tested No.	224	36	188	–
	Positive cases No. (%)	34 (15.18)	10 (27.78)	24 (12.77)	0.022
	Rate of HP tested/1,000 HP days	1.95	2.36	1.82	
	RR (CI_95%_)	-	1	0.77 (0.37–1.62)	0.49
	COVID-19 rate per 1,000 HP days	1.95	2.36	1.82	
	RR (CI_95%_)	–	1	0.77 (0.37–1.62)	0.562
Health personnel from COVID areas excluding NICU = 102	HP tested No.	112	18	94	–
	Positive cases No. (%)	20 (17.86)	8 (44.44)	12 (12.77)	0.0013
	Rate of HP tested/1,000 HP days	1.78	2.92	1.41	
	RR (CI_95%_)	–	1	0.48 (0.20–1.18)	0.104
	COVID-19 rate per 1,000 HP days	1.78	2.92	1.41	
	RR (CI_95%_)	–	1	0.48 (0.20–1.28)	0.061

^*^X^2^ tests were used to calculate p.

HP, Health Personnel; No., number; RR, relative risk; CI, confidence interval.

There was an overall decrease in the percentage of positivity in health personnel, from COVID areas and from COVID areas excluding the neonatal intensive care unit (NICU) in the second period (*p* < 0.03). Although the COVID-19/1,000 health personnel days rate decreased from one period to another, no statistically significant differences were found between the first and the second period, in any of the studied areas. We performed the analysis with and without the NICU, since this department never restricted the entrance to visitors.

**Figure 1:**
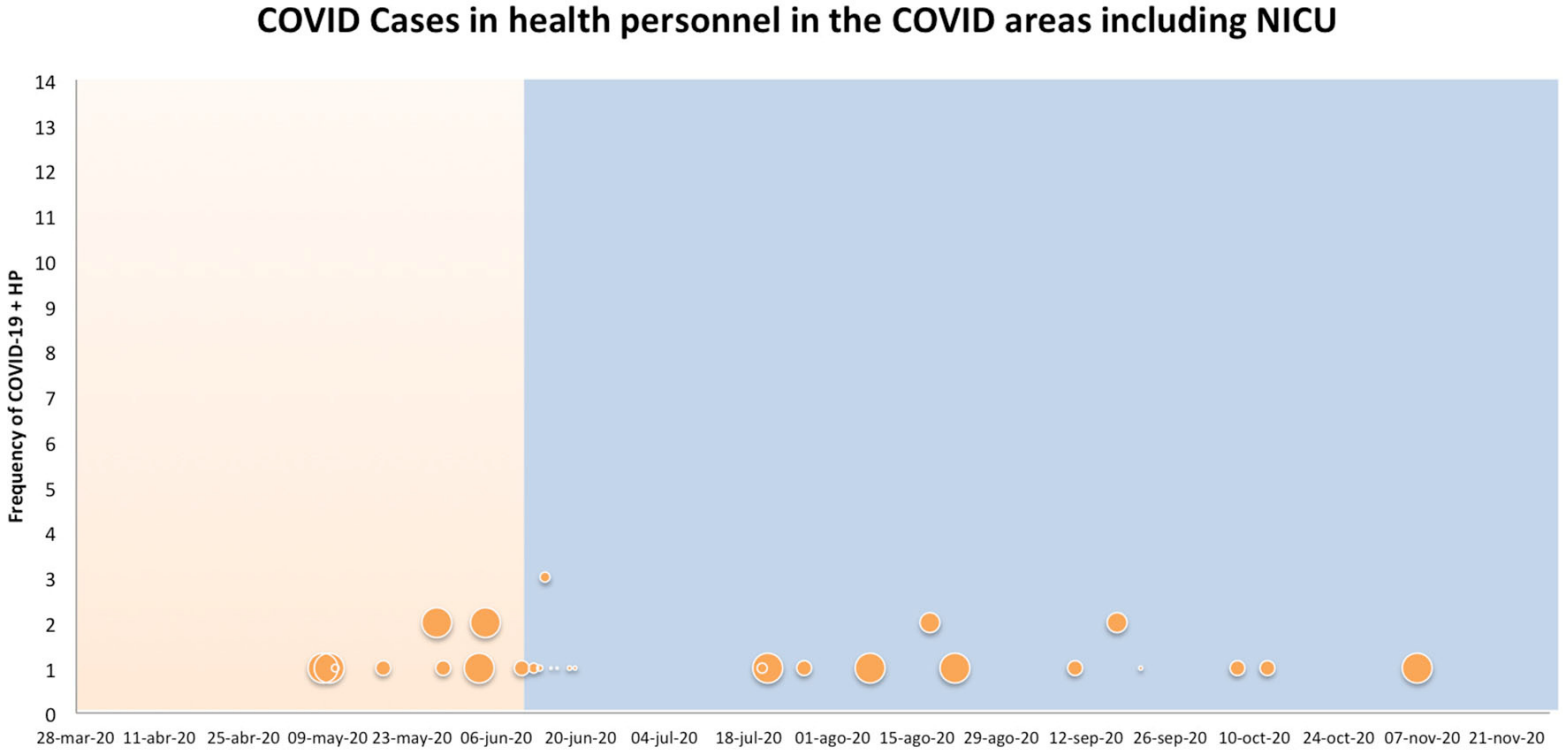
Orange and blue shades represent period 1 and 2 respectively. The size of the circles indicates the percentage of COVID-19 and HP tests. The large circles represent 100%, the medium circles 50% and the small ones 30%.

**Figure 2:**
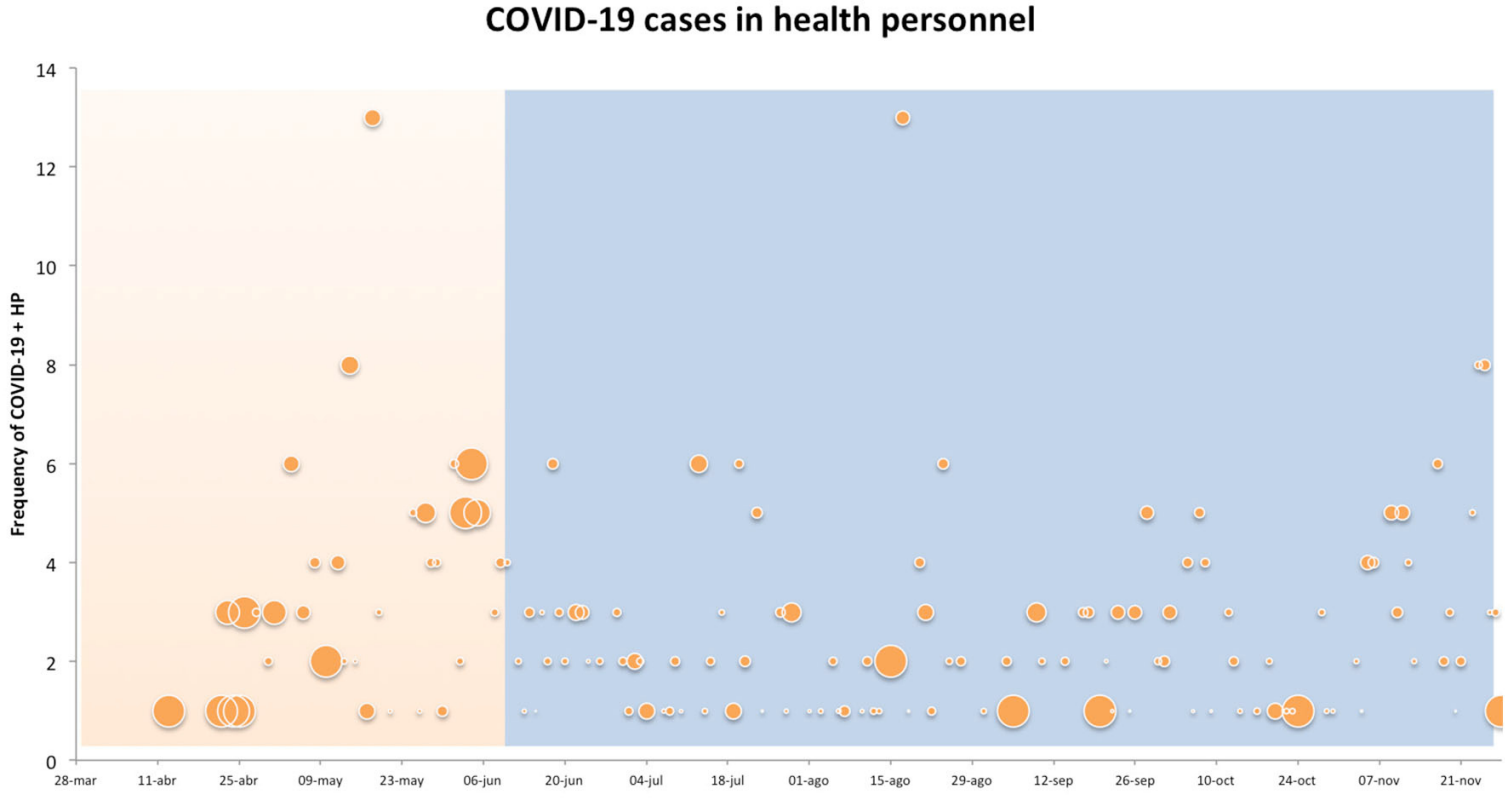
Orange and blue shades represent period 1 and 2 respectively. The size of the circles indicates the percentage of COVID-19 and HP tests. The large circles represent 100%, the medium circles 50% and the small ones 30%.

Figures 1, 2 show the frequency and positivity of the RT-PCR tests during the first and the second period of the study. Figure 1 shows the data from the COVID areas, while Figure 2 shows all the data from the hospital.

### Contagiousness in patients' relatives

A sub-analysis was carried out to determine the contagiousness among patients' relatives. During the time in which the study was conducted 216 patients with COVID-19 were treated at the hospital; although we tried to contact all the patients who left the COVID area, communication was only achieved with 145 (67.1%) patients, of whom seven died.

The median age of the patients was 7 years old (IQR 2–13.5). 52.4% were female and 47.6% were male. Most of the children were patients that had been under care and follow-up because of underlying conditions such as cancer (26.2%), neurological diseases (11.7%), gastrointestinal diseases (10.3%), blood disorders (9.7%), cardiac disorders (7.6%), and metabolic disorders (5.5%). Only 10 patients (6.9%) were previously healthy. The median COVID-19 area stay was 4 days (Annex 1).

During the first period, 16 children infected with COVID-19 were attended in the hospital. Parents/caregivers were not allowed to visit them, although they were asked daily about any symptoms or if they had tested positive to a COVID-19 test; none of the family members from those patients were infected. During the second period, six out of 129 (4.65%) parents/caregivers were infected, of which two (1.55%) were not evaluated to confirm the diagnosis. Three of them had no symptoms (2.33%), however they underwent the test because they were in contact with their child, and they had a positive test result. Only 1 (0.78%) had both signs and symptoms as well as a positive test. A total of 4 (3.1%) relatives had a positive test, although it cannot be confirmed that they got the infection while visiting their children. In all of the cases, either the symptoms or the positive tests appeared between 2 and 4 days after the patient's admission to the COVID-area. Two of the cases were in September 2020 and the rest of them were in January 2021. Those patients, whose parents became infected shortly after their hospitalization, spent an average of 3.8 (1–10) days hospitalized in the COVID area. This average is very similar to the 4 days spent by those patients whose parents were not infected. There were no statistically significant differences between the days of hospitalization of patients whose parents were infected and those whose parents were not infected (*p* = 0.39). Most of the patients had an underlying disease, that requires hospitalization, and also COVID-19. Therefore, the average hospital stay in COVID-area was 4 days, with a minimum of 1 day and a maximum of 21. Upon discharge, they were instructed to remain in isolation for 10 days from the onset of symptoms. We did not find differences in COVID-19 transmission according with hospitalization length.

## Discussion

Isolating pediatric COVID-19 patients was met with great resistance from the patients' families, and increasing, palpable anxiety and angst among children and their parents/caregivers; therefore, it appeared that allowing for supervised, controlled visits while closely monitoring the parents'/caregivers' health status would be a feasible alternative. In this study we were able to demonstrate that allowing parents/caregivers to visit using a standardized protocol, does not increase the rate of infections in health personnel and does not have a significant impact in spreading the infection to the children's families. It is important to consider that the longer the hospitalization time of patients, the greater the risk of infection for parents/caregivers. However, between parents/caregivers that became infected and those who did not, there were no statistically significant differences in the number of days their children were hospitalized.

The rate of COVID-19 cases among health personnel did not change, although the percentage of COVID cases among health personnel decreased during the second period of the study. This could be explained by several facts: When comparing the percentage of positivity in the HIMFG and in Mexico City, the pattern is very similar to the health personnel patterns, regardless of the periods in which parents/caregivers were allowed or not allowed to visit. In Mexico City the percentage of positive cases during the first period was 49.7%−54.4% and in period 2 was 21.5%−41.8% (26). Another reason for which there could be fewer cases, could be that over time, health personnel became more aware of the symptoms of COVID-19 and chose to be tested only if they had mild symptoms, while at the beginning of the pandemic many of the symptoms were unknown or unnoticed. On the other hand, when parents/caregivers helped with the care of patients, they performed some of the children care procedures that involved a higher exposure to body fluids (feces, saliva, mucus) (27, 28) potentially decreasing the risk of infection for health personnel. In addition to this, most children had an underlying medical condition, probably their parents/caregivers were more careful in hand washing and hygiene practices than other parents/caregivers. Also they were trained in hand hygiene to be able to enter as visitors. It is important to remember that, during the time of the study, there were no COVID-19 vaccines yet.

It is of note that an analysis of the COVID areas was done without considering the NICU because of two reasons: criteria for parental visits did not change in that department at the beginning of the pandemic and the NICU is on the top floor of the hospital and physically separated from all other areas. In both analyses, all COVID-19 areas and COVID-19 areas without the NICU, there were no significant differences in the risk of contagion before and after the entry of visitors.

This study demonstrates that with proper care and training, the spread of COVID-19 infection among parents/caregivers who visit their children can be controlled, as 95.4% were not infected. It is not possible to know if the parents/caregivers who became infected did so inside or outside the hospital, it is clear that they were at risk of contagion because they were within the COVID area, however it is also true that there was an increase in cases in Mexico City at the time that the infections occurred in the six parents/caregivers (September, December and January). It is possible that they could have acquired the infection in the community, as it follows the epidemic pattern of the city. Our findings support the benefit of pediatric patients to be accompanied (29–31) with low or inexistent harm for their families. To our knowledge, this is one of the first studies that analyzes the potential spread of infection from hospitals visits, and was not able to prove any risk increase from the visits. However to verify this information, future studies are required, especially studies in which patients, their parents/caregivers and health personnel are followed up over time and with larger sample sizes.

Virani et al. (11) mentions that there are different ethical dilemmas around whether to allow visits in pediatric hospitals during the COVID-19 pandemic. He argues that hospitals must ensure the health of patients, personnel and visitors, and that in terms of justice, the same visiting rules should be applied for everyone. He suggests promoting virtual visits (11), however in developing countries this is not feasible. In our study the same rules were taken in terms of infection prevention. Nevertheless in our hospital 70% of the patients are in a state of extreme poverty and there are constant failures with the Internet connection, therefore, is difficult to make routine virtual visits. In this study, remarkably, even in this condition of poverty and associated low educational level, it was possible to educate parents/caregivers to avoid infections. This can be extrapolated to similar contexts, and it is reasonable to expect that in populations with higher educational level the results would be at least as good.

Several authors have shown the increment in mental health issues in hospitals where no visits were allowed in COVID areas (8–10): A study carried out in Singapore by Fan et al. conclude that isolation affects both patients and family members and therefore strategies should be sought to identify and reduce stress and anxiety (9). In this study the psychology staff noticed that before implementing the protocol there were some children with psychological problems, some of them severe, such as suicide ideation and posttraumatic stress. In addition, some parents/caregivers showed aggressive behavior toward the hospital staff. Although these findings require further research, they noticed that after allowing parents/caregivers visits, these kind of problems almost disappeared.

Shonkoff has conceptualized the stress produced by being in a situation that the patient experiences as threatening, with perception of danger, as toxic stress. This type of stress carries neurophysiological and psychological problems that compromise the children's quality of life and psycho-emotional integrity (32, 33).

In our study, patients who were separated from their primary caregiver had severe consequences in the short and medium term: High levels of anxiety and symptoms compatible with acute and post-traumatic stress, which required psychological and psychiatric treatment for several months after discharge (research data will be presented in a future publication).

Regardless of the age of development, we can infer that those patients aged 5–17 years who presented these manifestations have greater chances of progressing in their development with fewer psycho-emotional consequences than those patients whose ages range from 6 months to 5 years, although long-term follow-up would be necessary to determine this impact. However, it has already been described in the literature that the consequences of separating children from their caregiver are usually irreversible if not treated promptly (32, 33).

A century's worth of evidence concludes that there is a negative effect on children when they are isolated from their primary caregiver with the damage being extended to parents/caregivers as well. Currently we already have strong evidence on the minimal risk of contagion that exists when allowing a family member to enter the COVID area. Although there is a risk that parents could become infected, with the training received, the percentage of infections was minimal and the benefit of letting them visit their children was greater. We agree with Kitano et al. who mention that “standarizing visitor restriction policies in pediatric hospitals by making a risk-benefit balance between optimizing family-centered care and decreasing potential sources of transmission of COVID-19, is challenging.” They suggest evaluating the effectiveness of visiting restrictions in terms of infection control and we consider that this study is a step in such evaluation (34). It is our responsibility, as professionals, to take care of the comprehensive health of our patients, so it is essential that the pediatric COVID area be regulated for this to happen. Therefore, the “do no harm” dictum also applies when preventing children from being separated from their loved ones.

There was also risk that parents/caregivers visiting their children with COVID-19 could become infected and spread the infection in the community. Determining this risk was outside the scope of the study. However, in one hand we have the following facts: only 6 parents became infected, the infection occurred during a wave peak, and all the infections could also have been acquired in the community. In the other hand there is vast and enough evidence of the negative effects of isolating children from their parents/caregivers. Therefore we consider that the benefit of implementing the protocol outweighs the risk.

Pines et al. reported that during the pandemic in the pediatric emergency department the visits were reduced in 59% for COVID-19 and no COVID-19 cases, even if they were allowed to visit: “Parents may potentially have avoided necessary care for their children” during hospitalization and the children's prognosis could have worsened (35). The fact of the continuous close care that parents/caregivers provide to children should be also emphasized in the guidelines that allow visitation to children, in order to reinforce the importance of visiting children with COVID-19.

This study has several limitations mostly related to the patients' relatives, since there were not enough financial resources to perform active surveillance requesting PCR test results to all the patients' relatives. We solely relied on symptom reports, making it impossible to ascertain that there were no other infected relatives that could have been asymptomatic. However, follow-up calls were made to the parents/caregivers and they were asked if they were other members of the family with symptoms, if any family member had tested positive for any tests they had done on their own and if they had been seriously ill or hospitalized for COVID-19. Of those contacted, none reported having had family members with severe illness or hospitalized. In addition, of the 216 patients with COVID-19, only 145 were contacted as some of them did not wanted to answer the questions, did not leave any contact numbers or left incorrect ones. Another limitation is that we cannot be sure of the source of the infection from the parents/caregivers: Family members could be infected from their hospitalized children or from anyone else outside the hospital. Additionally, of the 6 reported cases from this study, 4 occurred in January 2021, which coincides with an increase in cases within Mexico City, making even more difficult to define whether they were infected inside or outside the hospital. Another possible scenario is that parents/caregivers in the COVID area may have had an asymptomatic infection and may have been the source of infection for other parents.

In conclusion, this study demonstrates that allowing parents/caregivers or caregivers into COVID areas with the described protocol does not increase the spread of infection among health personnel. Also, with proper training, parents/caregivers or caregivers do not get infected and they barely spread the virus into their homes. Taking into consideration the plausible psychological and development consequences of isolating children and the absence of risk of infection in health personnel, as well as the proper training of caregivers, to allow parents/caregivers to visit their children seems to be the best strategy for the children's health during COVID-19. To develop and implement policies to permit the children to be accompanied during their hospitalization should be a standard in the context of an epidemic of COVID-19, which most likely can be safely carried to the endemic stage.

## Data availability statement

The raw data supporting the conclusions of this article will be made available by the authors, without undue reservation.

## Ethics statement

Ethical review and approval was not required for the study on human participants in accordance with the local legislation and institutional requirements. Written informed consent for participation was not required for this study in accordance with the national legislation and the institutional requirements.

## Author contributions

DR, MA-V, and AG-D: elaboration of the theoretical framework, search of bibliographical references, data analysis, interpretation and analysis of results, discussion and conclusion. ML-C, FO-R, SB-P, and CJ-E: protocol planning and implementation. CJ-E: elaboration of the theoretical framework, search of bibliographical references and data analysis. IR-T: implementation of the protocol and review of the manuscript. SO-R: carrying out patient censuses, caring for parents/caregivers in the COVID-19 area, follow-up calls to find out if there were infections 15 days after the patients were discharged. SS-S: writing and revising the manuscript. MV-G and JN-Z: protocol planning and review of the manuscript. LJ-B: patient review, data collection, writing and review of the manuscript. All authors contributed to the article and approved the submitted version.

## Funding

The study was fully funded by the Children's Hospital of Mexico. There was no financing from any other company or foundation.

## Conflict of interest

The authors declare that the research was conducted in the absence of any commercial or financial relationships that could be construed as a potential conflict of interest.

## Publisher's note

All claims expressed in this article are solely those of the authors and do not necessarily represent those of their affiliated organizations, or those of the publisher, the editors and the reviewers. Any product that may be evaluated in this article, or claim that may be made by its manufacturer, is not guaranteed or endorsed by the publisher.

## Appendix

**Annex 1:** Patient's baseline characteristics.

	***n* = 145**
Age – years Median (Interquartile range)	7 (2–13.5)
Female sex No. (%)	76 (52.4)
Type of condition prior to COVID 19 No. (%)
Cancer	38 (26.2)
Neurological	17 (11.7)
Gastrointestinal	15 (10.3)
Blood disorders	14 (9.7)
Cardiac	11 (7.6)
Previously healthy	10 (6.9)
Metabolic disorders	8 (5.5)
Kidney diseases	7 (4.8)
Pulmonary diseases	5 (3.4)
Genetic	5 (3.4)
Malformations	3 (2.1)
Infectious diseases	1 (0.7)
Psychiatric	1 (0.7)
Others	8 (5.5)
Days in COVID area median (interquartile range)	4 (2–8)

## References

[1] Suárez V Suarez Quezada M Oros Ruiz S Ronquillo De Jesús E. Epidemiología de COVID-19 en México: del 27 de febrero al 30 de abril de 2020 [Epidemiology of COVID-19 in Mexico: from the 27th of February to the 30th of April 2020]. Rev Clin Esp. (2020) 220:463–71. 10.1016/j.rce.2020.05.007 33994571 PMC7250750

[2] Maldonado A Reyes M. Distribution of confirmed with COVID-19 by age and gender in Mexico. medRxiv. (2021) 1–9. 10.1101/2021.11.21.2126409234057188

[3] González-García N Castilla-Peón2 MF Santos FS Jiménez-Juárez RN Bustamante MEM Hibert MAM. Covid-19 incidence and mortality by age strata and comorbidities in Mexico City : a focus in the pediatric population. Front Public Health. (2021) 9:1–6. 10.3389/fpubh.2021.738423 34568267 PMC8459904

[4] Barranco R Ventura F. Covid-19 and infection in health-care workers: an emerging problem. Med Leg J. (2020) 88:65–6. 10.1177/0025817220923694 32441196

[5] Chang MC Hur J Park D. Strategies for the prevention of the intra-hospital transmission of covid-19: a retrospective cohort study. Healthcare. (2020) 8:1–8. 10.3390/healthcare8030195 32635290 PMC7551914

[6] Chou R Dana T Buckley DI Selph S Fu R Totten AM. Epidemiology of and risk factors for coronavirus infection in health care workers: a living rapid review. Ann Intern Med. (2020) 173:120–36. 10.7326/M20-1632 32369541 PMC7240841

[7] Antonio-Villa NE Bello-Chavolla OY Vargas-Vázquez A Fermín-Martínez C Márquez-Salinas A Bahena-López JP. Health-care workers with COVID-19 living in Mexico City: clinical characterization and related outcomes. Clin Infect Dis. (2020) 52:1–21. 10.1093/cid/ciaa1487 32986819 PMC7543362

[8] Abad C Fearday A Safdar N. Adverse effects of isolation in hospitalised patients: a systematic review. J Hosp Infect. (2010) 76:97–102. 10.1016/j.jhin.2010.04.027 20619929 PMC7114657

[9] Fan T Shao L Wang X Ren P. Efficacy of copper-impregnated hospital linen in reducing healthcare-associated infections: a systematic review and meta-analysis. PLoS ONE. (2020) 15:e0236184. 10.1371/journal.pone.0236184 32687517 PMC7371175

[10] Siddiqi H. To suffer alone: hospital visitation policies during COVID-19. J Hosp Med. (2020) 15:694–5. 10.12788/jhm.3494 32853145

[11] Virani AK Puls HT Mitsos R Longstaff H Goldman RD Lantos JD. Benefits and risks of visitor restrictions for hospitalized children during the COVID pandemic. Pediatrics. (2020) 146:e2020000786. 10.1542/peds.2020-000786 32430441

[12] Murray PD Swanson JR. Visitation restrictions: is it right and how do we support families in the NICU during COVID-19? J Perinatol. (2020) 40:1576–81. 10.1038/s41372-020-00781-1 32772051 PMC7414900

[13] Yuan R Xu Q Xi C Lou C Xie Z Ge Q. Psychological status of parents of hospitalized children during the COVID-19 epidemic in China. Psychiatry Res J. (2020) 288:112953. 10.1016/j.psychres.2020.112953 32302814 PMC7153530

[14] Bembich S Tripani A Mastromarino S Di Risio G Castelpietra E Risso FM. Parents experiencing NICU visit restrictions due to COVID-19 pandemic. Acta Paediatr. (2021) 110:940–1. 10.1111/apa.15620 33063339 PMC7675454

[15] Andrist E Clarke RG Harding M. Paved with good intentions: hospital visitation restrictions in the age of coronavirus disease 2019. Pediatr Crit Care Med. (2020) 21:E924–6. 10.1097/PCC.0000000000002506 32541371 PMC7314338

[16] Ludvigsson JF. Children are unlikely to be the main drivers of the COVID-19 pandemic – a systematic review. Acta Paediatr. (2020) 109:1525–30. 10.1111/apa.15371 32430964 PMC7280674

[17] Gudbjartsson DF Helgason A Jonsson H Magnusson OT Melsted P Norddahl GL. Spread of SARS-CoV-2 in the Icelandic population. N Engl J Med. (2020) 382:2302–15. 10.1056/NEJMoa2006100 32289214 PMC7175425

[18] Park Y Choe Y Park O Park SY Kim YM Kim J. Contact tracing during coronavirus disease outbreak, South Korea, 2020. Emerg Infect Dis. (2020) 26:2465–8. 10.3201/eid2610.201315 32673193 PMC7510731

[19] Villa-Guillén M Garduño-Espinosa J Herrera-Segura MG Moreno-Espinoza S de la Rosa-Zamboni D López-Martínez B. Restructuring of a pediatric hospital in the face of the covid-19 pandemic. Bol Med Hosp Infant Mex. (2021) 78:3–9. 10.24875/BMHIM.20000265 33226975

[20] Luque-Coqui M Adame-Vivanco MJ De La Rosa-Zamboni D Mendoza-Rodríguez P Campos-Gutiérrez M Campos-Ugalde S. Implementation of guidelines to integrate the caregiver as a coassistant of health-care personnel during the hospital stay of COVID-19 pediatric patients: adaptation in a Mexican public pediatric hospital. Bol Med Hosp Infant Mex. (2021) 78:102–9. 10.24875/BMHIM.20000256 33651786

[21] Solís-Cámara P Lara M Rosa M Jiménez M Rodríguez J. Factor structure of the GHQ-12. General health questionnaire in Mexican population. Salud Soc. (2016) 7:62–7. 10.22199/S07187475.2016.0001.00004

[22] *Contact Tracing for COVID-19 | CDC* [Internet]. Centers for Disease Control and Prevention. (2021). Available online at: https://www.cdc.gov/coronavirus/2019-ncov/php/contact-tracing/contact-tracing-plan/contact-tracing.html (acessed November 5, 2021).

[23] Public Health Guidance for Community-Related Exposure | CDC [Internet]. Available online at: https://www.cdc.gov/coronavirus/2019-ncov/your-health/quarantine-isolation.html?CDC_AA_refVal=https%3A%2F%2F (accessed November 8, 2021).

[24] Operational Considerations for Adapting a Contact Tracing Program to Respond to the COVID-19 Pandemic in non-US Settings [Internet]. Centers for Disease Control and Prevention (CDC) (2020). Available online at: https://www.cdc.gov/coronavirus/2019-ncov/global-covid-19/contact-tracing.html?CDC_AA_refVal=https%3A%2F%2Fwww.cdc.gov%2Fcoronavirus%2F2019-ncov%2Fglobal-covid-19%2Foperational-considerations-contact-tracing.html (accessed April 13, 2020).

[25] de la Rosa-Zamboni D Ortega-Riosvelasco F González-García N Valladares-Wagner JM Saldívar-Flores A Aguilar-Guzmán O. Tracing COVID-19 source of infection among health personnel in a pediatric hospital. Front Pediatr. (2022) 10:1–8. 10.3389/fped.2022.897113 35757120 PMC9218243

[26] *COVID-19 Tablero México - CONACYT - CentroGeo - GeoInt - DataLab* [Internet]. (2021). Available online at: https://datos.covid-19.conacyt.mx/ (accessed November 17, 2021).

[27] Davoudi-Kiakalayeh A Mohammadi R Pourfathollah AA Siery Z Davoudi-Kiakalayeh S. SARS-CoV2 in different body fluids, risks of transmission, and preventing COVID-19: a comprehensive evidence-based review. Int J Prev Med. (2020) 11:1–7. 10.4103/ijpvm.IJPVM_255_20 33042494 PMC7518359

[28] Li H Wang Y Ji M Pei F Zhao Q Zhou Y. Transmission routes analysis of SARS-CoV-2: a systematic review and case report. Front Cell Dev Biol. (2020) 8:1–11. 10.3389/fcell.2020.00618 32754600 PMC7365854

[29] Pang EM Sey R De Beritto T Lee HC Powell CM. Advancing health equity by translating lessons learned from nicu family visitations during the covid-19 pandemic. Neoreviews. (2021) 22:e1–6. 10.1542/neo.22-1-e1 33386310

[30] Jones-Bonofiglio K Nortjé N Webster L Garros D. A practical approach to hospital visitation during a pandemic: responding with compassion to unjustified restrictions. Am J Crit Care. (2021) 30:302–11. 10.4037/ajcc2021611 33870412

[31] Goga A Feucht U Pillay S Reubenson G Jeena P Madhi S. Parental access to hospitalised children during infectious disease pandemics such as COVID-19. S Afr Med J. (2020) 111:100–5. 10.7196/SAMJ.2021.v111i2.15388 33944717

[32] Shonkoff JP Garner AS Siegel BS Dobbins MI Earls MF McGuinn L. The lifelong effects of early childhood adversity and toxic stress. Pediatrics. (2012) 129:e232–46. 10.1542/peds.2011-2663 22201156

[33] Garner AS Shonkoff JP. Early childhood adversity, toxic stress, and the role of the pediatrician: translating developmental science into lifelong health. Pediatrics. (2012) 129:e224–31. 10.1542/peds.2011-2662 22201148

[34] Kitano T Piché-Renaud P-P Groves HE Streitenberger L Freeman R Science M. Visitor restriction policy on pediatric wards during novel coronavirus (COVID-19) outbreak: a survey study across North America. J Pediatr Infect Dis Soc. (2020) 9:766–8. 10.1093/jpids/piaa126 33090211 PMC7665605

[35] Pines JM Zocchi MS Black BS Carlson JN Celedon P Moghtaderi A. Characterizing pediatric emergency department visits during the COVID-19 pandemic. Am J Emerg Med. (2020) 41:201−4. 10.1016/j.ajem.2020.11.037 33257144 PMC7682424

